# Classification and Regression Tree Approach for Prediction of Potential Hazards of Urban Airborne Bacteria during Asian Dust Events

**DOI:** 10.1038/s41598-018-29796-7

**Published:** 2018-08-07

**Authors:** Keunje Yoo, Hyunji Yoo, Jae Min Lee, Sudheer Kumar Shukla, Joonhong Park

**Affiliations:** 1 Department of Civil and Environmental Engineering, Yonsei University, 50 Yonsei-ro, Seodaemun-gu, Seoul, 03722 South Korea; 2 Department of Earth and Environmental Engineering, Columbia University, New York, NY 10027 USA; 3 Department of Earth System Sciences, Yonsei University, 50 Yonsei-ro, Seodaemun-gu, Seoul, 03722 South Korea; 4 Department of Built and Natural Environment, Caledonian College of Engineering, Seeb, Sultanate of Oman

## Abstract

Despite progress in monitoring and modeling Asian dust (AD) events, real-time public hazard prediction based on biological evidence during AD events remains a challenge. Herein, both a classification and regression tree (CART) and multiple linear regression (MLR) were applied to assess the applicability of prediction for potential urban airborne bacterial hazards during AD events using metagenomic analysis and real-time qPCR. In the present work, *Bacillus cereus* was screened as a potential pathogenic candidate and positively correlated with PM_10_ concentration (*p* < 0.05). Additionally, detection of the *bceT* gene with qPCR, which codes for an enterotoxin in *B. cereus*, was significantly increased during AD events (*p* < 0.05). The CART approach more successfully predicted potential airborne bacterial hazards with a relatively high coefficient of determination (R^2^) and small bias, with the smallest root mean square error (RMSE) and mean absolute error (MAE) compared to the MLR approach. Regression tree analyses from the CART model showed that the PM_10_ concentration, from 78.4 µg/m^3^ to 92.2 µg/m^3^, is an important atmospheric parameter that significantly affects the potential airborne bacterial hazard during AD events. The results show that the CART approach may be useful to effectively derive a predictive understanding of potential airborne bacterial hazards during AD events and thus has a possible for improving decision-making tools for environmental policies associated with air pollution and public health.

## Introduction

Asian dust (AD) events, global dust transport events, have increased over the last 20 years due to global climate change and desertification^1–3^. East Asia is a major source region of global wind-blown dust aerosols. In spring and winter, dust uplifted from arid Asian areas is transported to northern China, Korea, Japan, and even as far as the western United States^1,2^. AD events are becoming less predictable due to an increase in the fraction of unanticipated dust particles derived from the newly formed deserts in western China and Mongolia^1,2^. Most previous studies have suggested that AD events result in increased occurrences of human diseases and environmental problems^1,2,4,5^. Therefore, AD events are recognized as a major social/environmental/clinical issue, with growing concern in East Asia^1^.

Although biological agents in AD have received scant attention compared with physiochemical attributes, there is increasing evidence that exposure to bioaerosols during AD events may cause adverse health effects and severe diseases when pathogenic bacteria are involved^2,6,7^. To investigate their effects on public health during AD events, an appropriate methodology must define potential pathogens and employ an effective monitoring system^8,9^; however, there is sparse information on urban airborne bacterial communities^2,9^. Next-generation sequencing (NGS) can offer insights into the diversity and composition of airborne culturable and non-culturable bacteria^7,10^. Research suggests that 16S rRNA gene-based NGS can successfully determine the abundance and diversity of potentially pathogenic bacteria for screening purposes in activated sludge, biosolids, drinking water, and soil^11–14^.

Identification of pathogens in bioaerosols requires long-term monitoring, and assessing bioaerosol risks to human health is time-consuming and costly. Instead, current real-time atmospheric environmental parameters are not only closely related to the occurrence of AD events but are also relatively faster and easier to analyze than detecting and assessing potential pathogens during AD events^15^. Therefore, modeling that depends on statistical analysis could be an alternative approach for exploring the relationship between airborne bacterial communities and atmospheric environmental conditions^16^. If certain relationships can be found between them it will then be possible to predict potential hazards one or two days in advance and more effectively protect public health^17,18^. Most importantly, reliable short-term prediction of potential airborne bacterial hazards may assist the authorities in managing atmospheric environmental policy for AD events. Despite the extensive research on physiochemical modeling studies during AD events^19,20^, no specific research has so far been carried out to predict biological hazards during AD events.

Multiple linear regression (MLR) is one of the widely used statistical tools for finding an appropriate mathematical model and for determining the best-fitting coefficients of a model from the given data^16,18^. MLR generally provides good predictive capability in environmental studies, such as air quality prediction models^16,18^, and can provide reasonable interpretation between dependent and predictor variables by statistical tests^21^. Machine learning and rule induction is a powerful statistical method for collecting, summarizing, and analyzing data from different perspectives into valuable and practical information to identify useful relationships^22,23^. As a representative machine learning method, the classification and regression tree (CART) has considerable advantages, including that it is nonparametric and is suitable for nonlinear structures and that it may be appropriate for solving complex, dynamic environmental problems from a small dataset^22,24^. Rule induction employed in CART can be used to find key rules on the basis of interactions between independent and dependent variables^22,23^. CART approaches have been used in environmental forecasting research to estimate urban air quality^18^, determine groundwater pollution vulnerability^24^, predict *in situ* dechlorination potential^25^, predict water quality from wastewater treatment plants^26^, assess microbial source tracking^27^, and predict heavy metal sorption to soil^28^. Therefore, CART and MLR models could support decision-making and effective management of potential urban airborne bacterial hazards during AD events. However, no detailed comparison of the model performance has yet to be evaluated.

The aims of this study are to (1) compare the predictive abilities between MLR and CART approach for assessing potential airborne bacterial hazards during AD events, and (2) identify key atmospheric environmental parameters that significantly influence potential airborne bacterial hazards during AD events.

## Results

### Characterization of Atmospheric Parameters between AD and Non-AD Events

The average PM_10_ concentration of AD events was 178 µg/m^3^, which was significantly (t-test, *p* < 0.001) higher, by 112 µg/m^3^, than that of non-AD events (Table 1). Seasonal monitoring revealed that airborne bacterial abundance with PM_10_ concentrations was more than 10- to 50-fold higher during AD events, and non-AD events did not affect airborne bacterial abundance. Although studies^5,6^ have indicated that atmospheric indicators such as temperature and relative humidity exhibit relatively high correlations during AD events, our monitoring results revealed no significant difference between AD and non-AD events. The parameters of the other air masses (e.g., wind speed, sunshine, evaporation, and surface temperature) displayed no differences between AD and non-AD events (Table 1).

**Table 1:** Statistical summary of the data for the atmospheric environmental parameters and 730 airborne bacterial parameters between AD events (n = 10) and non-AD events (n = 45).

Atmosphere environment parameters	AD events	Non-AD events	*p* value
PM_10_ (µg/m^3^)	178 ± 97	66 ± 25	<0.001
Temperature (°C)	12.9 ± 5.9	16.8 ± 10.3	
Relative humidity (%)	42.2 ± 10.2	55.8 ± 12.9	
Wind speed (m/s)	3.1 ± 0.6	2.8 ± 1.1	
Duration of sunshine (hr)	6.0 ± 1.8	8.0 ± 2.2	
Evaporation (mm)	3.7 ± 2.4	3.1 ± 1.7	
Surface temperature (°C)	15.5 ± 7.1	17.5 ± 10.9	
**Airborne bacterial parameters**	**AD events**	**Non-AD events**	***p*** **value**
Bacterial abundance (copy numbers/m^3^)	6.05E + 07 ± 1.00E + 06	3.22E + 05 ± 1.37E + 04	<0.001
Bacterial diversity (Shannon index)	4.21 ± 0.63	2.87 ± 0.41	
Relative abundance of potential pathogenic bacteria (%)	0.97 ± 0.32	0.55 ± 0.18	<0.05
Relative abundance of *B. cereus* group (%)	0.62 ± 0.18	0.19 ± 0.16	<0.05
*bceT* gene abundance (copy numbers/m^3^)	4.27E + 04 ± 3.15E + 03	2.26E + 03 ± 2.44E + 02	<0.05

The *p* values were calculated with t-test in SAS v. 9.2.

### Characteristics of Bacterial Communities between AD and Non-AD Events

The abundance of airborne bacteria was determined by qPCR, targeting the 16S rRNA gene in samples collected during the three study years. The 16 S rRNA gene copy numbers ranged from 4.85 × 10^3^ to 2.58 × 10^8^ gene copies/m^3^. During AD events, the gene copy numbers (mean: 6.05 × 10^7^ gene copies/m^3^, Stdev: 1.00 × 10^6^) increased remarkably compared to the non-AD (mean: 3.22 × 10^5^ gene copies/m^3^, Stdev: 1.37 × 10^4^) levels (*p* < 0.001) (Table 1). Additionally, the bacterial 16 S rRNA gene copy numbers tended to correlate positively with PM_10_ concentration (Supplementary Fig. S1a). As indicated by the Shannon index (H′) values, airborne bacterial diversity significantly increased during AD events (Supplementary Fig. S1b). The increased airborne bacterial diversity during AD events and correlation with dust parameters suggest that dust events increase local airborne bacterial diversity.

**Figure 1:**
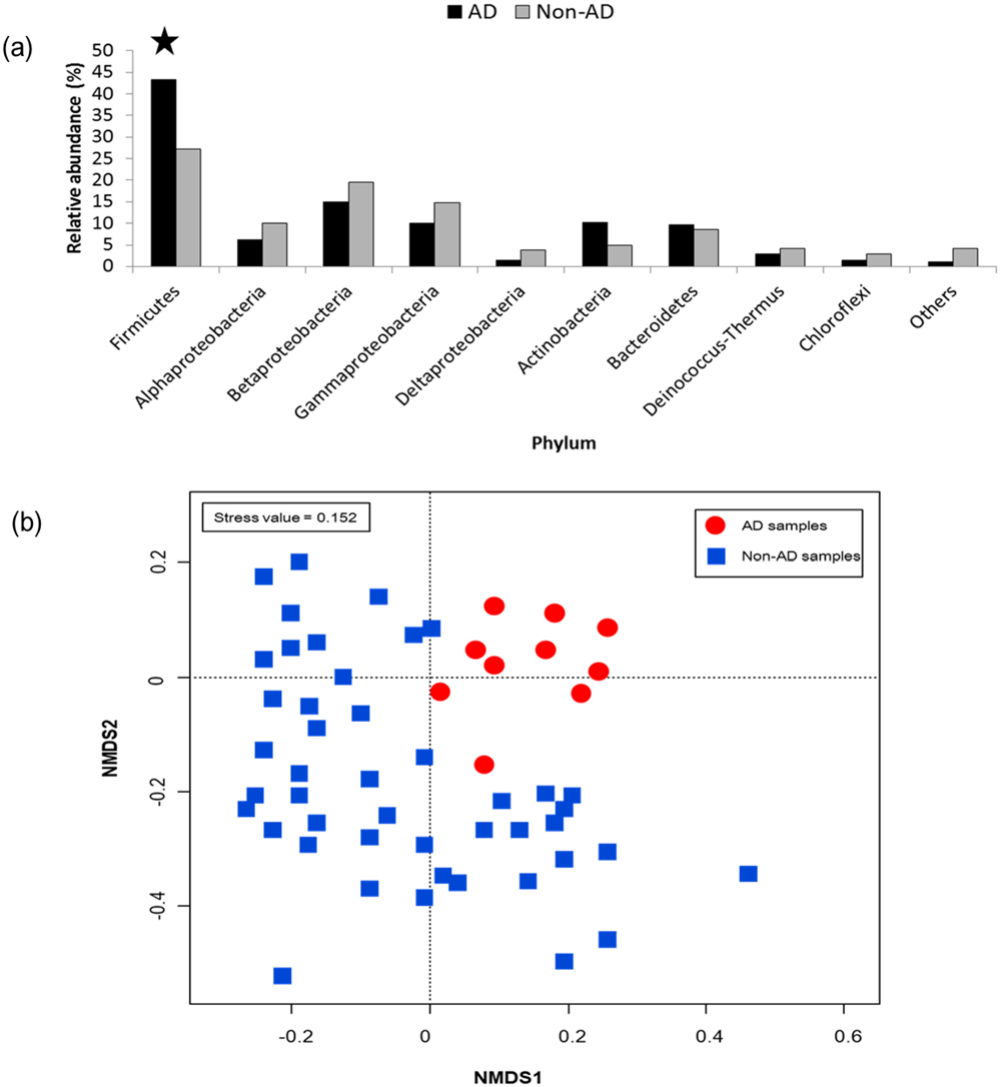
Relative abundance of airborne bacterial community structures between AD events and non-AD events (**a**) and non-metric multidimensional scaling (NMDS) ordination at the phylum level (**b**). Others indicate minor genus members with relative abundances <1.00%. *p < 0.05 (t-test in SAS v. 9.2).

AD and non-AD events were characterized by different bacterial taxa (Fig. 1). *Firmicutes* significantly increased with those for the non-AD events (*p* < 0.05) and composed the most dominant bacterial group during AD events (Fig. 1a). According to the NMDS plot, airborne bacterial structures of the AD samples were clustered together and separated from those of non-AD samples (Fig. 1b), indicating that AD events caused a significant shift in microbial community structures.

These results imply that although the nature of aerosol bacterial populations is variable, most airborne bacteria during AD events may be associated with particle size and air environmental conditions. A significant correlation between bacterial diversity and PM_10_ abundance during AD events suggested that desert dust might be the source of airborne bacteria^29^. According to the backward trajectory analysis (Supplementary Fig. S2), air masses during AD events contained microorganisms originating from the Gobi Desert that passed over China and the Yellow Sea to Seoul. However, air masses from non-AD events contained microorganisms transported from various directions near Korea. These results may support that the shift in airborne bacterial communities between AD and non-AD events is affected by the source of airborne bacteria and transport pathways (Supplementary Fig. S2).

### Screening of Potential Pathogenic Bacteria Candidates

**Figure 2:**
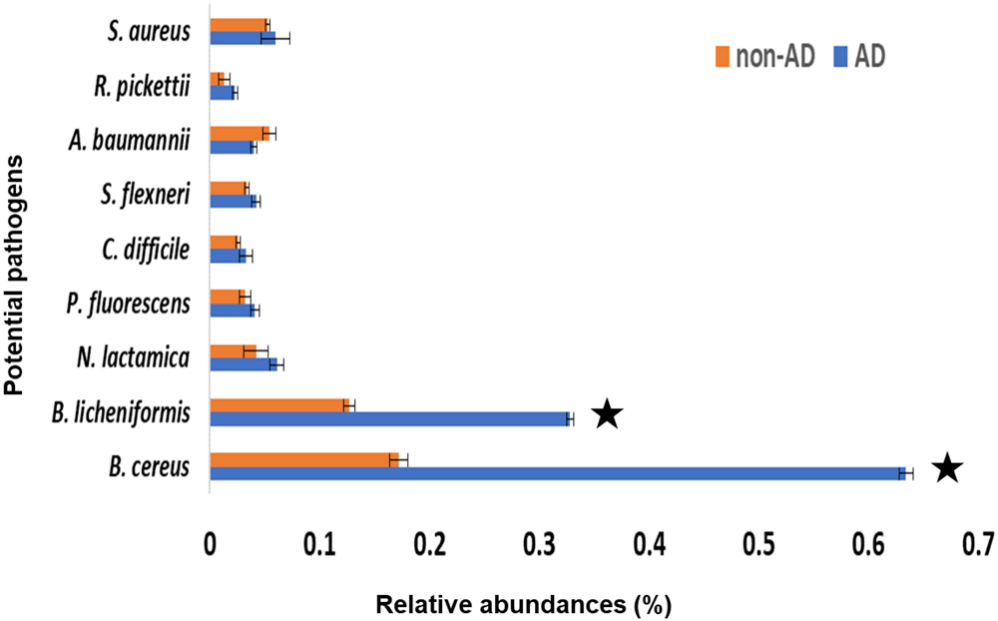
Relative abundance of potential pathogenic bacteria candidates among the total 16S rRNA gene sequence reads from the Pyrosequencing. * indicates *p* < 0.05 from t-test in SAS v.9.2.

The sequences obtained using pyrosequencing were extracted by alignment with reference sequences, and all sequences were assigned at the species level (Supplementary Table S1). Potential pathogenic bacteria belonging to *Bacillus, Neisseria, Pseudomonas, Clostridium, Shigella, Acinetobacter, Ralstonia*, and *Staphylococcus* were detected in non-AD samples (Fig. 2), suggestive of the potential presence of bacterial hazards in urban bioaerosol environments, even though the 16 S rRNA gene sequence is limited in its ability to accurately determine pathogenicity^13,30^. The relative abundance of potential pathogenic bacteria candidates increased significantly during AD events and was positively correlated with PM_10_ concentration (Supplementary Fig. S1c). Compared with non-AD samples, significantly higher *Bacillus* (a potential pathogenic candidate) was detected in AD samples. In particular, *B. cereus* and *B. licheniformis* significantly increased (*p* < 0.05), suggestive of their potential as AD-specific bacterial pathogen candidates (Fig. 2). Although *B. licheniformis* was identified as an AD-specific candidate pathogen, the primer information on its pathogenic gene is insufficient for quantitative examination. Conversely, however, sufficient primer information of the pathogenic gene for *B. cereus* has been established previously. Therefore, we selected *B. cereus* as the AD-specific candidate pathogen.

The abundance of *bceT* gene copy numbers ranged from 3.27 × 10^4^ to 1.15 × 10^5^ gene copies/m^3^ during AD events (Table 1). *BceT* gene copy numbers exhibited a similar trend as the relative abundance of potential pathogenic bacteria (Supplementary Fig. S1c) and were significantly higher during AD events (*p* < 0.05).

### Assessment of Prediction Performance for AD Events

After demonstrating that airborne bacterial parameters, in particular bacterial hazards, increased significantly (*p* < 0.05) during AD events, we used AD-specific airborne bacterial parameters to evaluate whether the MLR and CART models could achieve good performance in reflecting AD event characteristics. According to the performance indexes, the CART approaches outperformed the MLR approaches (Table 2). Most airborne bacterial parameters yielded good correlations between predicted and real-time measured values in the CART model (Table 2). The estimates of the relative abundance of potential pathogenic bacteria, *B. cereus* populations, and *bceT* gene abundance for AD events displayed relatively good fits (R^2^ = 0.71–0.77) with the least bias and smallest RMSE (11.3–14.4) and MAE (7.25, 10.4) in the test set results (Table 2). CART and rule induction effectively reproduced variations in airborne bacterial parameters using on-site measurement data, in particular the relative abundance of *B. cereus* populations and *bceT* gene abundance during AD events (Table 2).

**Table 2:** Performance indicators for the developed predictive MLR and CART models.

Target	Subset		Performance Indexes		
			RMSE	MAE	R^2^
Bacterial abundance (16S rRNA gene copies)	MLR	Training	8.67	7.43	0.76
		Test	15.7	12.2	0.68
	CART	Training	6.48	4.04	0.81
		Test	10.2	8.02	0.70
Bacteria diversity (Shannon index)	MLR	Training	15.4	10.7	0.65
		Test	23.3	15.9	0.58
	CART	Training	8.14	5.25	0.78
		Test	13.2	10.8	0.66
Relative abundance of potential pathogenic bacteria	MLR	Training	14.2	12.3	0.72
		Test	22.8	17.2	0.61
	CART	Training	9.01	5.87	0.78
		Test	14.4	10.4	0.71
Relative abundance of *B. cereus*	MLR	Training	18.4	12.6	0.70
		Test	26.1	19.2	0.58
	CART	Training	7.80	4.79	0.82
		Test	11.3	7.25	0.77
*bceT* gene abundance	MLR	Training	16.4	10.3	0.66
		Test	23.5	16.1	0.54
	CART	Training	8.48	6.04	0.78
		Test	12.3	9.07	0.75

### Identification of Important Variables Associated with Airborne Bacterial Parameters

The CART and rule induction method has outstanding advantages in terms of identifying independent variables that may significantly influence its dependent variables and in providing rule induction between the independent and dependent variables^23^.

**Figure 3:**
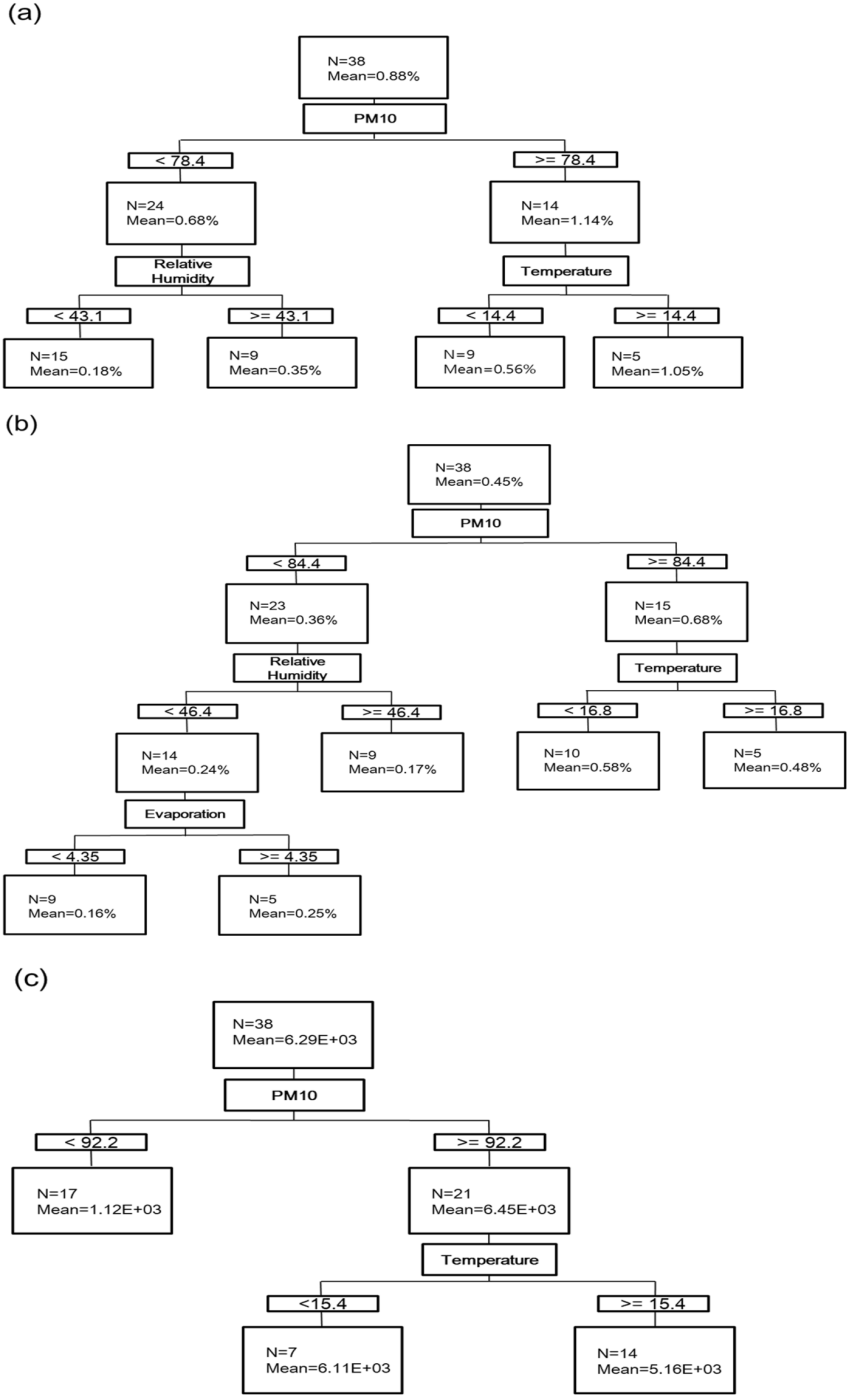
Determination of the relative importance of the predictor variables in the CART model for prediction of relative abundance of potential pathogens (**a**) and *B. cereus* (**b**), and *bceT* gene abundance (**c**) by binary regression tree analysis.

To induct a rule between the atmospheric environmental input variables and target variables (airborne bacterial parameters), we performed a CART-based tree analysis. The final regression trees generated by rule induction with the airborne bacterial parameters for each child node of this tree in the training dataset were shown (Fig. 3, Supplementary Fig. S3). With respect to the independent variables, the first split of the tree was defined as the PM_10_ subject (Fig. 3a). Fourteen datasets were clustered with PM_10_ concentrations ≥78.4 µg/m^3^, and the remaining twenty-four datasets were clustered with PM_10_ concentrations <78.4 µg/m^3^. Higher PM_10_ subjects were segregated based on the temperature subject (Fig. 3a). Figure 3b was constructed for the relative abundance of *B. cereus* as predictors. The first split of the tree was defined with respect to the PM_10_ subject, and the nodes were segregated with relative humidity and temperature as the subject (Fig. 3b). All figures can be interpreted in the same way (Fig. 3, Supplementary Fig. S3). A relative importance ranking of individual parameters for airborne bacterial hazards was possible (Supplementary Table S2). PM_10_, relative humidity, and temperature took precedence over the other parameters and were deemed essential parameters for predicting the airborne bacterial hazard potential.

## Discussion

Recently, the East Asian region’s climatic conditions such as scarce rains and droughts have boosted the persistence of atmospheric bioaerosols^1^. Therefore, it is important to integrate this process into air quality modeling systems intended for air quality planning and assessment in order to assess impacts on human health^31^ and ecosystems^32^. Although it is recognized that dust particles contain pathogens, in most cases the potential hazards or risks associated with them is still largely unclear^2^. The pathogenic bacteria effect of dust inhalation can be attributed to the direct physical action of dust particles, and may be exacerbated by the toxic effects of biologically active compounds^33^. Although prediction accuracy was overall good as shown our study (Table 2), regression models such as MLR have certain limitations. For example, it is relatively difficult to reflect non-linear conditions, and multi-collinearity between independent and dependent variables usually causes MLR to be inefficient^32^. Motivated by knowledge of these limitations, we applied the CART and rule induction method to predict potential hazards of urban airborne bacteria during AD events. This CART and rule induction approach successfully evaluated the prediction performance between observed, real-time measurable atmosphere environmental parameters and airborne bacterial parameters from NGS-based screening and targeted toxin genes from qPCR results. These results could be because the training datasets fit relatively well, reflecting the relationships between airborne bacterial parameters and atmospheric environmental parameters. From these results, we suggest that the correlations between airborne bacterial parameters and atmospheric environmental parameters during AD events are an approximately good fit with the CART and rule induction method for predicting the potential bacterial hazard in urban areas. Although the 16S rRNA gene sequence has been restricted to identifying the taxonomic resolution of bacterial pathogens^13,30^, combining high-throughput sequencing and qPCR results can provide relatively high resolution^34^. Because metagenomic approaches could be used to screen potential pathogens in AD samples, the identified potential pathogens subsequently could be quantified by using qPCR, which targets the potential pathogens using their biomarkers^34^.

During AD events, biological concentrations significantly increase with PM_10_ concentrations, with differences in bacterial community structure. The high correlation of bacterial abundances with PM_10_ during the AD events (Table 1, Supplementary Fig. S1) and backward trajectory results (Supplementary Fig. S2) in this study indicate that desert dust might be the source of airborne bacteria. However, there were not significant changes during non-AD events. These results indicate that the high concentration of bacteria during AD events was due to the large increase of the concentration of soil-originated particles which contained higher bacterial concentration^1–3^. The airborne bacteria from AD events may have mixed with indigenous airborne bacterial communities before reaching our sampling point, having traveled through industrial, agricultural, and urban areas^5,6^. As such, the suspended particle composition (e.g., PM_10_) may have been affected due to the addition of local pollutants and physicochemical changes in the atmospheric environment during transport; therefore, the frequency of potential pathogenic bacteria may have increased during AD events, which could affect ecosystem and human health. PM_10_ always segregated the first split of the tree, while temperature, relative humidity, and evaporation were important in predicting the airborne bacterial parameters in the rule induction (Fig. 3, Supplementary Fig. S3). PM_10_ is well established as an indicator of heavy air pollution, based on physical and chemical results and clinical evidence^35^. There is mounting evidence of the negative effects of bioaerosols associated with PM_10_ on ecosystems and human health^36,37^. However, the correlation between airborne bacterial parameters, including potential pathogens, and PM_10_ in urban areas during AD events is not well understood.

From our results, high PM_10_ concentrations were significantly correlated with potential pathogen indicators during AD events (Table 1, Supplementary Fig. S1). When the training datasets were constructed to predict bacterial abundance and diversity in the CART model, most PM_10_ concentrations were segregated into two split nodes between 65.3 and 70.8 µg/m^3^ (Supplementary Fig. S3). Meanwhile, the relative abundances of potential pathogens, *B. cereus*, and the *bceT* gene were segregated into higher PM_10_ concentrations (78.4 to 92.2 µg/m^3^) than bacterial abundance and diversity (Fig. 3), suggesting that the relative abundances of potential pathogens, *B. cereus*, and *bceT* gene were more significantly affected by PM_10_ concentrations and AD events than seasonal changes and local environmental effects. Our results revealed PM_10_ concentrations between 78.4 and 92.2 µg/m^3^ during AD events, indicative of a relatively high risk. PM_10_ prediction has attracted special legislative and scientific attention due to its negative effects on human health^38^. Since these results could offer AD-specific bacteria or relative environmental parameters for the implementation of a robust biosurveillance network, current air pollution policy may be further improved by taking into consideration the potential of biological hazards during AD events.

Airborne bacteria growth is affected by relative humidity and temperature^39^. Temperatures above 24 °C decrease airborne bacterial survival^39^, while relative humidity of 70–80% has a protective effect on aerosolized bacteria^40,41^. The temperature during most AD events (13–17 °C) may have supported airborne bacteria survival; however, the relative humidity (40–50%) may have adversely affected survival. The CART approach reflected the characteristics of these heterogeneous atmospheric conditions during AD events better than descriptive statistics, and successfully identified key atmospheric parameters associated with AD events and airborne bacteria. Thus, although aerosol bacterial populations are variable, the airborne bacteria community during AD events might be associated with specific atmospheric conditions.

Endospore-forming bacteria (e.g., *Bacillus*) have been isolated from inter-continentally transported dust^2,42,43^. These high-tolerance bacteria could survive during long-range dispersal and be efficiently transported by atmospheric dust^1,2^, shielded from inactivation by ultraviolet light and low relative humidity by attaching to crevasses within coarse particles. The trajectories pathway (Supplementary Fig. S2) is also considered to represent a protective mode that allows for the survival of *B. cereus* in hostile environments. Numerous fungal, bacterial, and viral species have been found in desert dust samples^2,42^. Endotoxins and other biologic compounds in PM_10-2.5_ from dust storms can activate inflammatory responses^44,45^. For example, in North Carolina ambient PM_10-2.5_ exacerbated allergic response to airborne bacteria^44^, and in six European cities the PM_10-2.5_ fraction triggered the highest inflammatory effect^45^.

The correlation between bacterial abundance and particulate matter in the air is likely a result of the dependence of bacteria on coarse particles (e.g., PM_10_) rather than on fine particles (e.g., PM_2.5_)^46^. Thus, molecular airborne bacteria community data with PM_10_ characteristics is rational to investigate the distribution and changes in airborne bacterial communities during AD events by resolving genetic diversity and populations. There are two reasons for excluding the possibility of a correlation between airborne bacterial communities and PM_2.5_. First, a large amount of PM_2.5_ are basically produced via homogeneous processes in the atmosphere, with no direct association with pre-existing particles^47^. Second, the suggested correlation is potentially wrong, since coarse and fine particles are not significantly correlated, according to the *Murata and Zhang*^46^ study. There are usually primary particles among PM_2.5_ such as fine particles, and the increase of coarse particles such as PM_10_ is commonly accompanied with an increase in fine particles in East Asia. This is supported by the dependence of airborne bacteria on dust particles^5,43^.

This study quantified the independent effects of different PM_10_ fractions, included a large distribution of complete differences among PM_10_ concentrations on case and control days, which provided acceptable statistical significance to detect relative high or low significant effects, with minimizing misclassification. Although machine learning and rule induction from small data sets makes the modeling procedure difficult and prone to overfitting, there are many situations in which organizations must work with small data sets in environmental analysis^48^. Thus, it is worthwhile to start developing appropriate forecasting models with smaller variance of forecasting error and good accuracy based on small data sets. To avoid overfitting due to the use of the small data set, k-fold cross-validation and random sampling alternatively can be used in the CART model^23,49^. Previous studies reported that k-fold cross-validation and random sampling are useful when no test sample is available and the learning sample is too small to have the test sample removed from it^49,50^. Although we tried to decrease error and biased predictors, relatively small-sized training and test data still can result in overfitting or misclassifications in this study. Therefore, further validation of our results is needed. Because recent studies have suggested that resampling and virtual data generation significantly improved predictive accuracy^48,51^, resampling and virtual data generation can be considered as an alternative method to improve problems inherent within small data sets. Additionally, if a sufficiently large dataset were obtained to further test the feasibility of this approach, the concepts outlined in this study could have potentially broad applications in real-time forecasts. Our concept can be potentially useful for further designing the spatial distribution of monitoring networks to protect public health during AD events. In addition, it could provide a scientific reference for the policy maker in developing future policies.

## Material and Methods

### Bioaerosol Sample Collection

We collected 55 air samples from 2011 to 2013 in Seodaemun-gu of Seoul, Korea, of which 16 were from the rooftop of the Seoul Air Monitoring Station in Bulgwang (37°61′31″N, 126°93′01″E) in 2011, and 39 were from the rooftop of the 3^rd^ Engineering building of Yonsei University in Shinchon (37°33′42″N, 126°56′07″E) in 2012 and 2013. These sites are located about 10 km from each other in an urban area characterized by human activities without industrial complexes. All air samples were collected 20–30 m above the ground. Ten AD events occurred in Seoul, Korea in 2011 and 2013. All data were separated into AD (ten samples) and non-AD (45 samples) events based on the “Asian Dust Occurrence Reports” from the National Institute of Environmental Research (NIER), Korea.

Bioaerosol samples were collected with a high-volume air sampler (Thermo Scientific, MA, USA). Samples were collected for 24 h at air flow rates of 300–500 L/min on 8 × 10-in. track-etched polycarbonate membrane filters (0.2 µm pore size; Whatman, GE, USA). The filters were autoclaved before sampling, and the filter holder in sampling apparatus was cleaned with 70% ethanol before each sampling event to avoid microbial contamination. After sampling, each filter was stored at −20 °C before DNA extraction.

### DNA Extraction from Bioaerosol Samples

Genomic DNA was extracted using a Fast DNA spin for Soil Kit (MPBiomedicals, OH, USA) following a previous method^52^, with slight modifications^15^. A negative control was included with every set of DNA extractions. These negative controls were treated exactly the same as all the samples through the entire experiment process, including amplification and sequencing. The extracted DNA samples were stored at −20 °C until use.

### Total Bacterial and bceT gene Quantification in Bioaerosol Samples

The total numbers of bacterial 16S rRNA genes copied from each bioaerosol sample were measured using qPCR with an iQ5 Real-Time PCR Detection System (Bio-Rad, CA, USA). The total reaction volume was 20 µL, containing 1× SYBR Master Mix (Bio-Rad), primer sets (300 nM each), and 10-fold-diluted template DNA. The primers targeting bacterial 16S rRNA gene and *bceT* gene have been described previously^53,54^. Because *bceT* is the pathogenic gene in *B. cereus*, and usually causes illness through the production of enterotoxin^55^, we used it to quantitatively examine the presence of potential pathogenic bacteria. A total of 1 × 10^1^ to 1 × 10^7^ copies/reaction of PCR products of *Escherichia coli* W3110 and *Bacillus cereus* strain KACC 11240 were used as the standard DNA template to generate a standard curve to quantify the 16S rRNA and *bceT* genes. For 16S rRNA gene, the thermal cycling conditions were followed as: 94 °C for 10 min, followed by 40 cycles at 94 °C for 15 s and 60 °C for 60 s. For *bceT* gene, the thermal cycling conditions were followed as: 95 °C for 5 min, followed by 37 cycles of 95 °C for 10 s and 60 °C for 45 s. Gene copy numbers (per m^3^) were calculated as described previously^56^. For the qPCR run of each sample, triplicate reactions were performed with positive and negative controls. Melting curve analysis (Tm) was performed for 1 cycle of 95 °C for 15 s, 1 cycle of 60 °C for 20 s and 1 cycle from 60 °C to 95 °C for 20 min.

### NGS Targeting Bacterial 16S rRNA Gene in Bioaerosol Microbial Communities

In this study, 454 FLX pyrosequencing was used to characterize microbial communities between AD and non-AD events. To provide PCR amplicons for the pyrosequencing, 563 F/16 (5′-AYTGGGYDTAAAGNG-3′) and BSR926/20 (5′-CCGTCAATTYYTTTRAGTTT-3′) targeting V4-V5 regions of 16S rRNA gene were amplified as described previously^57^. Forward primers included pyrosequencing adapter sequences and 8-bp barcode to distinguish each sample in the pool of amplicons^15^. PCR was conducted with a C1000TM Thermal Cycler (Bio-Rad) as follows: 3 min for 94 °C, followed by 35 cycles of 94 °C for 1 min, 55 °C for 30 s, 72 °C for 1 min, and a final extension at 72 °C for 5 min^15^. Negative controls consisting of the same process were included in each PCR run. Amplicons were pooled at equal concentrations using a NanoDrop 1000 spectrophotometer (Thermo Fisher Scientific, Wilmington, DE, USA), and PCR purification was performed using the MinElute PCR Purification Kit (Qiagen, CA, USA). Pyrosequencing was performed on a 454 GS-FLX Titanium Instrument (Roche, NJ, USA) at Macrogen (Seoul, Korea).

Quality control and taxonomic analysis of the 16S rRNA gene sequence reads were performed with Mothur package v.1.30 according to Schloss’ SOP^58^. All sequencing analysis process was performed following our previous work^15^. The obtained sequences were separated according to the barcodes, and quality filtering was performed using the Flowgram filtering method. Low-quality sequences with more than one mismatch to the barcode, two mismatches to the primer, or ambiguous nucleotides, negative controls were discarded. Sequences were removed if the homopolymers were longer than 8 bps and/or sequences were shorter than 300 bps^59^. UCHIME was used to remove expected chimeras derived from PCR using chimera.uchime from Mothur^60^. To remove or reduce PCR amplification and sequencing errors, sequences were denoised using the shhh.seqs command in AmpliconNoise in Mothur^61^. After quality filtering, sequences were aligned with the SILVA reference database using the NAST algorithm^58,62^, and similar sequences (≥97% similarity) were clustered into operational taxonomic units (OTUs). Sequences were assigned to phylotypes using the RDP classifier^63^. Non-metric multidimensional scaling (NMDS) was performed using the vegan package in R to visualize the taxonomic structure differences between AD and non-AD samples. The data were based on the Bray–Curtis dissimilarity measure of the binary matrix information of 55 air samples.

To screen for human pathogenic bacteria sequence candidates, representative 16S rRNA gene sequences of the bacterial genera OTUs were matched with the reference list of 16S rRNA gene sequences for known human pathogenic bacteria (Supplementary Table S1) from existing databases and studies^11,13,64^ using BLAST (blastn, cut-off identity ≥97%)^65^, and the first-cut screened sequences were matched again (identity >97%) using EzTaxon^66^ to identify bacterial 16S rRNA gene sequences similar to those of known pathogenic isolates.

### Characteristics of Atmosphere Environmental Parameters

Daily atmospheric environmental parameter measurements were obtained from the NIER, Korea (http://www.airkorea.or.kr/) using fully automated and daily measurements of atmospheric environmental parameters (e.g., PM_10_, temperature, relative humidity, wind speed, duration of sunshine, evaporation, and surface temperature). Available atmospheric environmental parameter data were extracted from the NIER daily, and averaged over the sampling time. Where data were missing for particular atmospheric environmental parameters on a given day, the values from the remaining data were used to compute the average. Daily information was provided by the Korea Meteorological Administration (KMA) (http://web.kma.go.kr/eng/index.jsp). Descriptive statistics were calculated for each parameter using SAS v.9.2 (SAS Institute Inc., USA).

### Data Processing of Multiple Linear Regression and CART

Multiple linear regression (MLR) is one of the most widely used methodologies for modeling the dependence of a dependent variable on several independent variables^17^. In general, a linear regression model assumes that (a) the error term has a normal distribution with a mean of 0, (b) the variance of the error term is constant across cases and independent of the variables in the model and (c) the value of the error term for a given case is independent of the values of the variable in the model and of the values of the error term for other cases.

MLR is one of the modeling techniques to investigate the relationship between a dependent variable and several independent variables^17,18^. In the MLR model, the error term denoted by *ε* is assumed to be normally distributed with mean 0 and variance *σ*^2^ (which is a constant). *ε* is also assumed to be uncorrelated. Thus, the regression model can be written as^17^:

1 $$y={b}_{0}+\sum _{i=1}^{n}{b}_{i}{x}_{i}+\varepsilon $$

where b_*i*_ are the regression coefficients, *x*_*i*_ are independent variables and *ε* is stochastic error associated with the regression. To estimate the value of the parameters, the least squares method was used.

CART is a nonparametric statistical technique developed by *Breiman et al*.^23^ that can solve classification and regression problems for categorical and continuous dependent variables. One notable advantage is that the models are scalable to large problems and small datasets^23^. CART is constructed by subsets of a dataset using all predictor variables to repeatedly create two child nodes beginning with the entire dataset^23^, and uses a stepwise method to establish splitting rules^23^. Although there are seven single variable splitting criteria, the Gini index is the default method, and it usually performs best^23^.

We included seven properties (PM_10_, temperature, relative humidity, wind speed, duration of sunshine, evaporation, and surface temperature) as independent variables and five properties (bacterial abundance, bacterial diversity, relative abundance of potential pathogenic bacteria, *B. cereus*, and *bceT* gene) as dependent variables in MLR and CART model. In CART, the Gini index was used to determine the dataset. To evaluate model performance, we partitioned the data into training (70% of the dataset for each class) and testing (remaining 30% of the entire dataset) datasets. The training dataset was used to find an optimal value from one or more predictors during the CART model construction. The testing dataset was used to evaluate the optimal value by verifying the prediction accuracy of the dependent variables. We used the SAS for the MLR model learning and SAS Enterprise Miner v.9.2 (SAS Inc.) for the CART model learning. Ten-fold cross-validation was used to avoid model over-fitting^23,67^. In this study, the data randomly broke into ten different parts. We used nine of these parts to train the model and the remaining part to test the model performance. We repeated these nine more times, using each of the ten parts as testing data. Then, we averaged the accuracy of the model in classifying the testing samples over each of the ten datasets to obtain a measure for the accuracy of MLR and CART.

### Model Performance Criteria

We evaluated the performance of the constructed MLR and CART model statistically, using the root mean square error (RMSE), mean absolute error (MAE), and coefficient of determination (R^2^)^18^ to evaluate the MLR and CART model performance between the dependent variables and predicted values of the response. Each performance criteria term indicates specific information regarding the predictive performance efficiency^18^. RMSE is a quadratic scoring rule that measures the average magnitude of the error. It gives a relatively high weight to large errors; hence, it is most useful when large errors are undesirable^18^. MAE measures the average magnitude of the error in a set of predictions without considering their direction. It is a linear score, implying that all individual differences between predictions and corresponding observed values are weighted equally in the average^18^. R^2^ is the best single measure of how well the predicted values match the observed values^18^. RMSE, MAE, and R^2^ are defined by the equations:

2 $${\rm{RMSE}}=\sqrt{\frac{{\sum }_{i=1}^{n}{({Q}_{pre}-{Q}_{obs})}^{2}}{n}}$$

3 $${\rm{MAE}}=[\frac{{\sum }_{i=1}^{n}|{Q}_{pre}-{Q}_{obs}|}{n}]$$

4 $${R}^{2}=[1-\sum _{i}\frac{{({Q}_{obs}-{Q}_{pre})}^{2}}{{({Q}_{obs}-{\bar{Q}}_{obs})}^{2}}]$$

where Q_*obs*_ = observed value; ${\bar{{\rm{Q}}}}_{obs}$ = the mean of the observed data; Q_*pre*_ = predicted value; *i* = number of observations; and *n* = number of points in the dataset. The best score for RMSE and MAE is defined as minimizing the training error; the measure is 1 for R^2^ and 0 for the other measures.

## Electronic supplementary material

Supplementary material
