# Supplementary material for: Classification and Regression Tree Approach for Prediction of Potential Hazards of Urban Airborne Bacteria during Asian Dust Events
Source: Sci Rep. 2018 Aug 7;8:11823. doi: 10.1038/s41598-018-29796-7 (PMC6081373; doi:10.1038/s41598-018-29796-7)
Supplement: Supplementary file 1 — Supplementary material [file 41598_2018_29796_MOESM1_ESM.docx]

**Classification and Regression Tree Approach for Prediction of Potential Hazards of Urban Airborne Bacteria during Asian Dust Events**

**Keunje Yoo^1,2*^, Hyunji Yoo^1^, Jae Min Lee^3^, Sudheer Kumar Shukla^4^, Joonhong Park^1, *^**

**^1^ Department of Civil and Environmental Engineering, Yonsei University, 50 Yonsei-ro, Seodaemun-gu, Seoul, South Korea**

**^2^ Department of Earth and Environmental Engineering, Columbia University, New York, NY 10027, USA**

**^3^ Department of Earth System Sciences, Yonsei University, 50 Yonsei-ro, Seodaemun-gu, Seoul, South Korea**

**^4^ Department of Built and Natural Environment, Caledonian College of Engineering, Sultanate of Oman**

Number of pages - 10

Number of figures - 3

Number of tables - 2

**Classification and regression tree (CART)**

CART method uses tree-building algorithms, which are a set of if then (split) conditions that permit prediction or classification of cases (Breiman et al., 1984; Berry and Linoff, 2004). A CART method that predicts the value of continuous variables from a set of continuous and/or categorical predictor variables is referred as regression type model. One noticeable advantage of CART is that the decision tree based models are scalable to large problems and can handle smaller data set (Marcham et al, 2000).

The CART method involves the segregation of different values of classification variables through progressive binary splits. Every value of each predictor variable is considered as a potential split, and the optimal split is selected based on impurity criterion (the reduction in the residual sum of squares due to a binary split of the data at that tree node). When missing values are encountered in considering a split, they are ignored and the probability and impurity measures are calculated from the non-missing values of those variables. Each parent node in the decision tree produces two child nodes, which in turn can become parent nodes producing additional child nodes (Figure). This process continues with both tree building and pruning until statistical analysis indicates that the tree fits without over-fitting the information contained in the data set. As a result, CART analysis produces trees that are simple to interpret and may be applied at the bedside.

CART is a nonparametric procedure that uses a stepwise method to establish splitting rules (Breiman et al., 1984). It includes seven single variable splitting criteria, namely Gini, Twoing, Ordered Twoing, Class Probability for classification trees, Least Squares, and Least Absolute Deviation for regression trees, and also one multivariable splitting criterion, the Linear Combinations method. Gini index is the default method that usually performs best, but under specific circumstances, other methods may also perform better. Gini index searches for the largest category in the dataset and strives to isolate it from the other categories (Breiman et al., 1984).


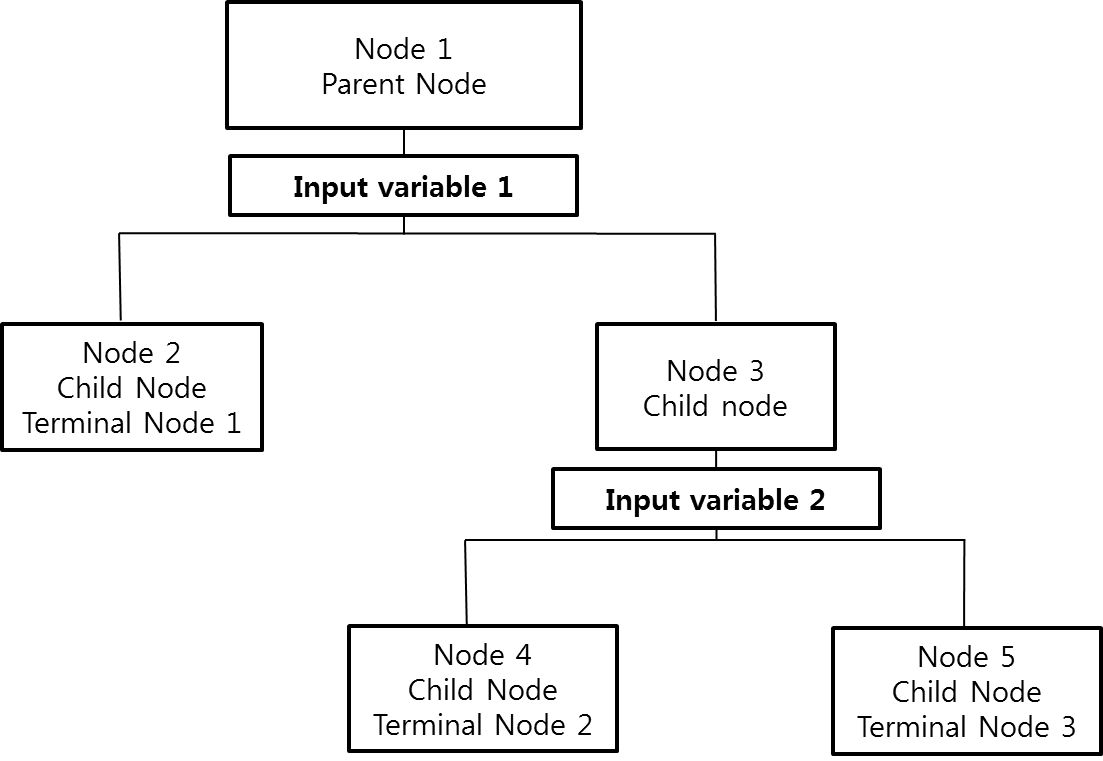


<Structure of binary tree from CART>

**References**

Breiman, L., Friedman, J., Olshen, R., Stone, C. 1984. Classification and Regression Tree. Champman and Hall, New York.

Berry, M., Linoff, G., 2004. Data Mining Techniques. Indianapolis, Indiana, USA, Wiley publishing, Inc.

Markham, I. S., Mathieu, R. G., Wray, B. A., 2000. Kanban setting through artificial intelligence: A comparative study of artificial neural networks and decision trees. *Integrated Manufacturing*, 11, 239-246.

**Table S1.** Reference list of genus and species of acknowledged human pathogens in this study (GenBank)

| Genus | species |
| --- | --- |
| *Acinetobacter* | *baumannii* |
| *Actinobacillus* | *All species* |
| *Actinomyces* | *bovis, israelii, naeslundii, pyogenes* |
| *Aeromonas* | *hydrophila, punctata* |
| *Amycolata* | *autotrophica* |
| *Arcanobacterium* | *haemolyticum* |
| *Arizona* | *hinshawii* |
| *Bacillus* | *anthracis, cereus, licheniformis* |
| *Bartonella* | *bacilliformis, henselae, quintana, vinsonii* |
| *Bordetella* | *pertussis, parapertussis* |
| *Borrelia* | *recurrentis* |
| *Brucella* | *abortus, canis, melitensis, ovis, suis* |
| *Burkholderia* | *mallei, pseudomallei* |
| *Calymmatobacterium* | *granulomatis* |
| *Campylobacter* | *coli, fetus, jejuni* |
| *Chlamydia* | *psittaci, trachomatis, pneumoniae* |
| *Clostridium* | *botulinum, chauvoei, difficile, haemolyticum, histolyticum, novyi, perfringens, septicum, tetani* |
| *Corynebacterium* | *bovis, jeikeium, diphtheria, pseudotuberculosis, renale, ulcerans* |
| *Coxiella* | *burnetii* |
| *Dermatophilus* | *congolensis* |
| *Edwardsiella* | *tarda* |
| *Erysipelothrix* | *rhusiopathiae* |
| *Escherichia* | *coli O117:H7* |
| *Fusobacterium* | *necrophorum* |
| *Francisella* | *tularensis* |
| *Haemophilus* | *ducreyi, influenzae* |
| *Helicobacter* | *pylori* |
| *Klebsiella* | *All species* |
| *Legionella* | *All species* |
| *Leptospira* | *interrogans* |
| *Listeria* | *monocytogenes* |
| *Moraxella* | *All species* |
| *Mycobacterium* | *africanum, asiaticum, avium, bovis, chelonae, fortuitum, kansasii, leprae, malmoense, marinum, paratuberculosis, scrofulaceum, simiae, szulgai, tuberculosis, ulcerans, xenopi* |
| *Mycoplasma* | *All species* |
| *Neisseria* | *gonorrhoeae, meningitides, sicca* |
| *Nocardia* | *asteroides, brasiliensis, farnicica, otitidiscaviarum, transvalensis* |

Table S1 Continued

| **Genus** | **Species** |
| --- | --- |
| *Orientia* | *tsutsugamushi* |
| *Pasteurella* | *haemolytica, multocida, pneumotropica* |
| *Plesiomonas* | *shigelloides* |
| *Pseudomonas* | *aeruginosa* |
| *Rickettsia* | *akari, australis, canada, conorii, japonica, montana, parkeri, prowazekii, rhipicephali, rickettsia, siberica, typhi* |
| *Rhodococcus* | *equi* |
| *Salmonella* | *All species* |
| *Shigella* | *dysenteriae, boydii, flexneri, sonnei* |
| *Staphylococcus* | *aureus* |
| *Streptobacillus* | *moniliformis* |
| *Streptococcus* | *agalactiae, pneumoniae, pyogenes* |
| *Treponema* | *carateum, pallidum, pertenue* |
| *Vibrio* | *cholerae, parahaemolyticus, vulnificus* |
| *Yersinia* | *pestis, enterocolitica, pseudotuberculosis* |

**Table S2.** Relative importance of the atmospheric parameters used to construct the representative classification tree of airborne bacterial hazard potential

|  | **Atmospheric parameter** | **Relative importance** |
| --- | --- | --- |
| Relative abundance of potential pathogenic bacteria | PM_10_ | 1.000 |
|  | Relative humidity | 0.432 |
|  | Evaporation | 0.374 |
|  | Temperature | 0.253 |
|  |  |  |
| Relative abundance of  *B. cereus* | PM_10_ | 1.000 |
|  | Relative humidity | 0.563 |
|  | Temperature | 0.474 |
|  | Wind speed | 0.241 |
|  |  |  |
| *BceT* gene abundance | PM_10_ | 1.000 |
|  | Temperature | 0.584 |
|  | Relative humidity | 0.472 |

* Relative importance scores were determined with the CART algorithm based on the usefulness of variables over all possible splits

**
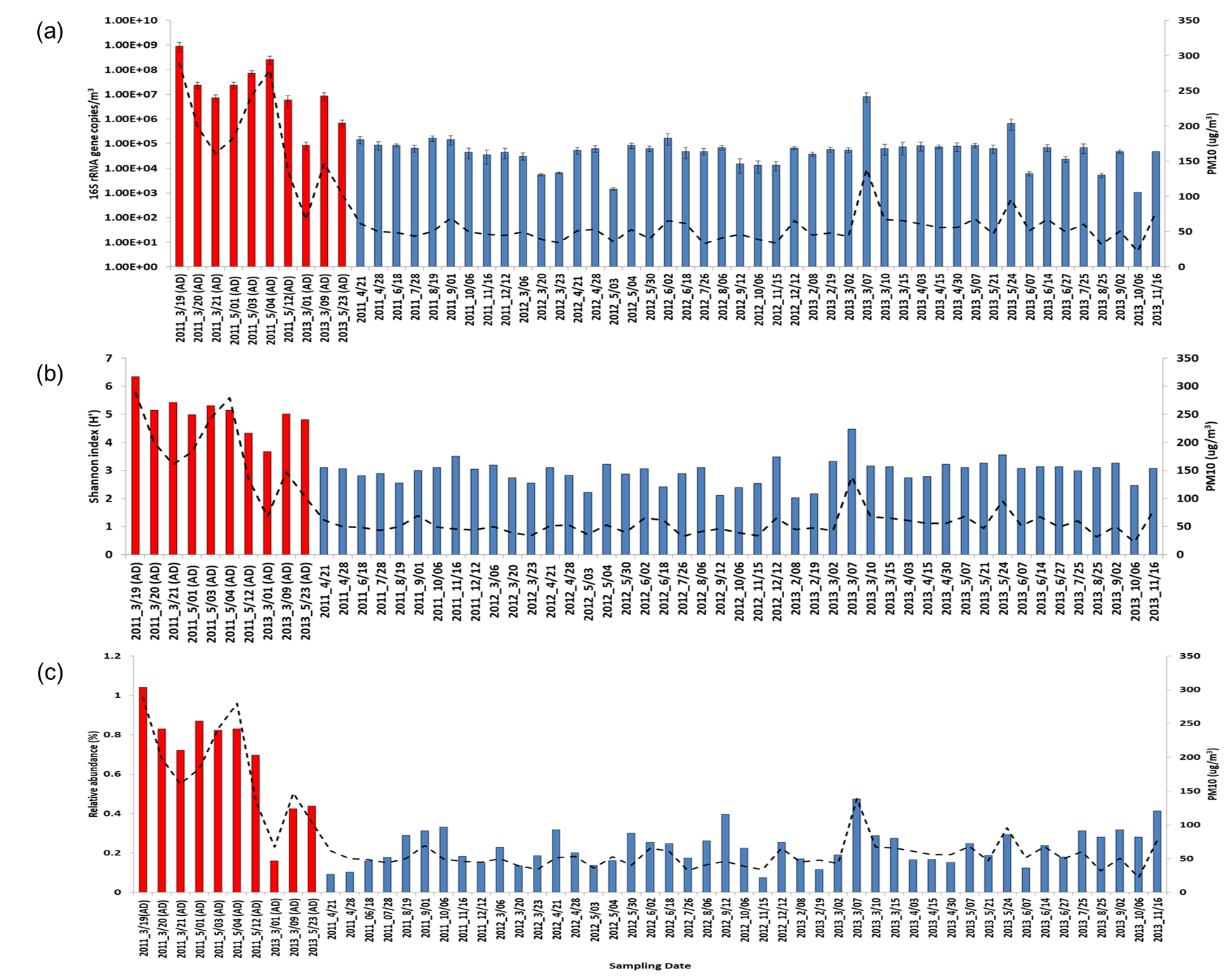
**

**Figure S1.** (A) 16S rRNA gene based real-time qPCR results for total bacteria according to PM_10_ concentrations during three years of monitoring. (B) Bacterial diversity from air samples was estimated with the Shannon index according to PM_10_ concentrations during three years of monitoring. (C) Relative abundances of potential pathogenic bacteria were estimated according to PM_10_ concentration during three years. Bars and error bars represent the mean and standard deviation, respectively. The qPCR results are expressed on a log scale to better describe the low detection level.


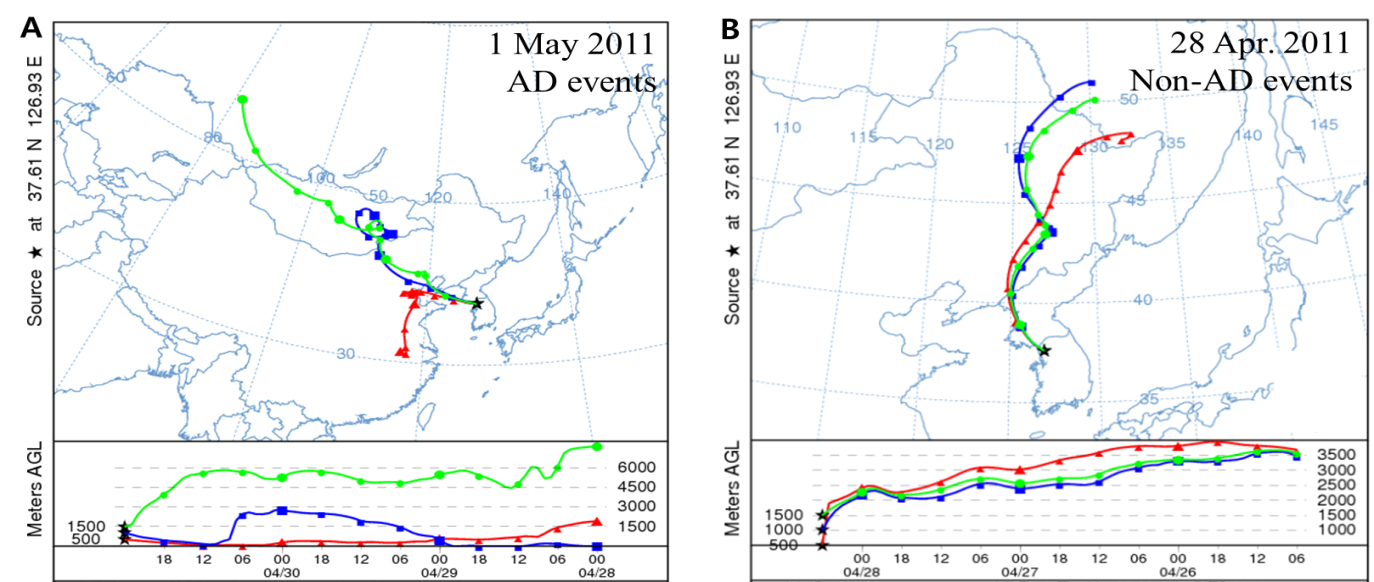


**Figure S2.** The NOAA HYSPLIT backward trajectory was analyzed to determine the route taken by air masses at sampling stations during (A) AD events, and (B) non-AD events from 500, 1000, and 1500 m. Backward trajectory analyses were conducted during 72 h and obtained from the NOAA ARL online service.


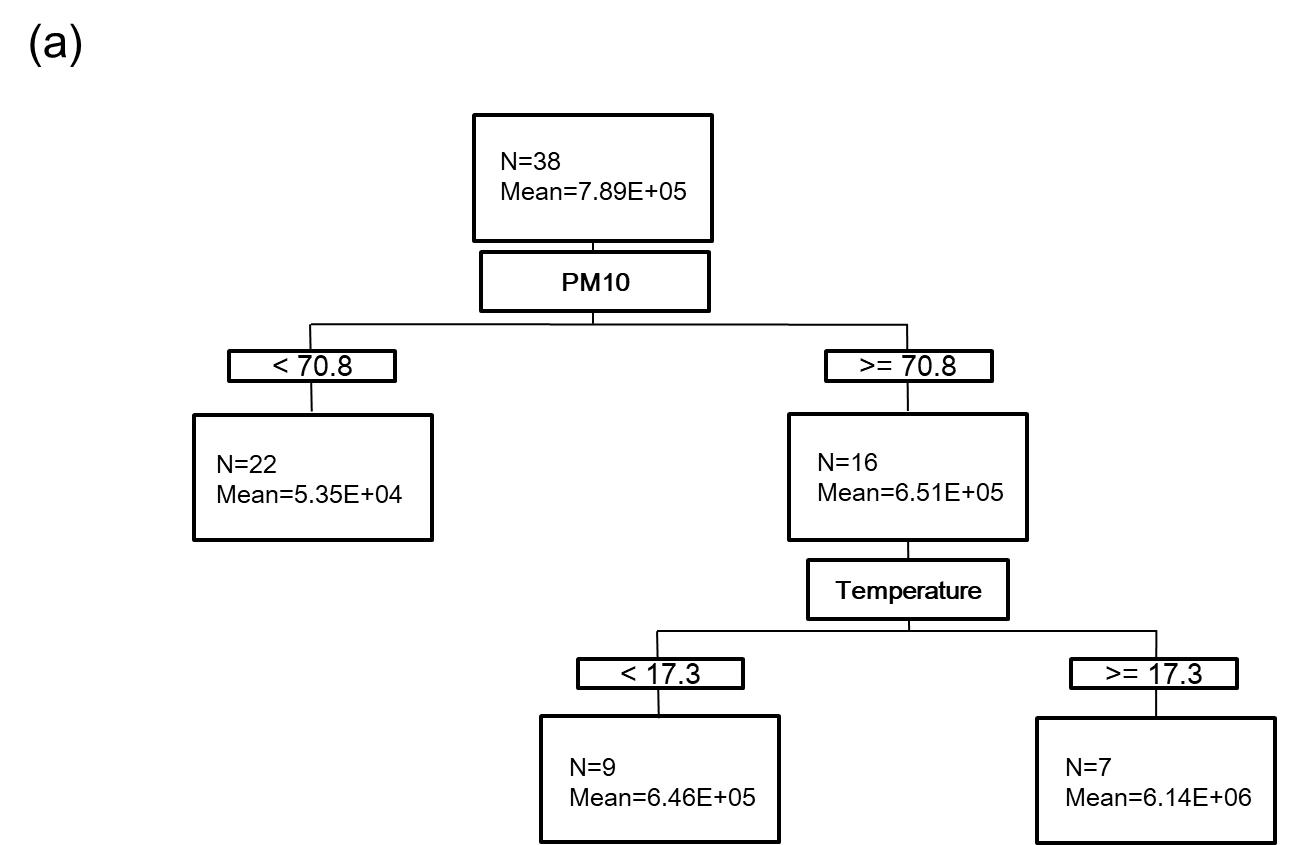


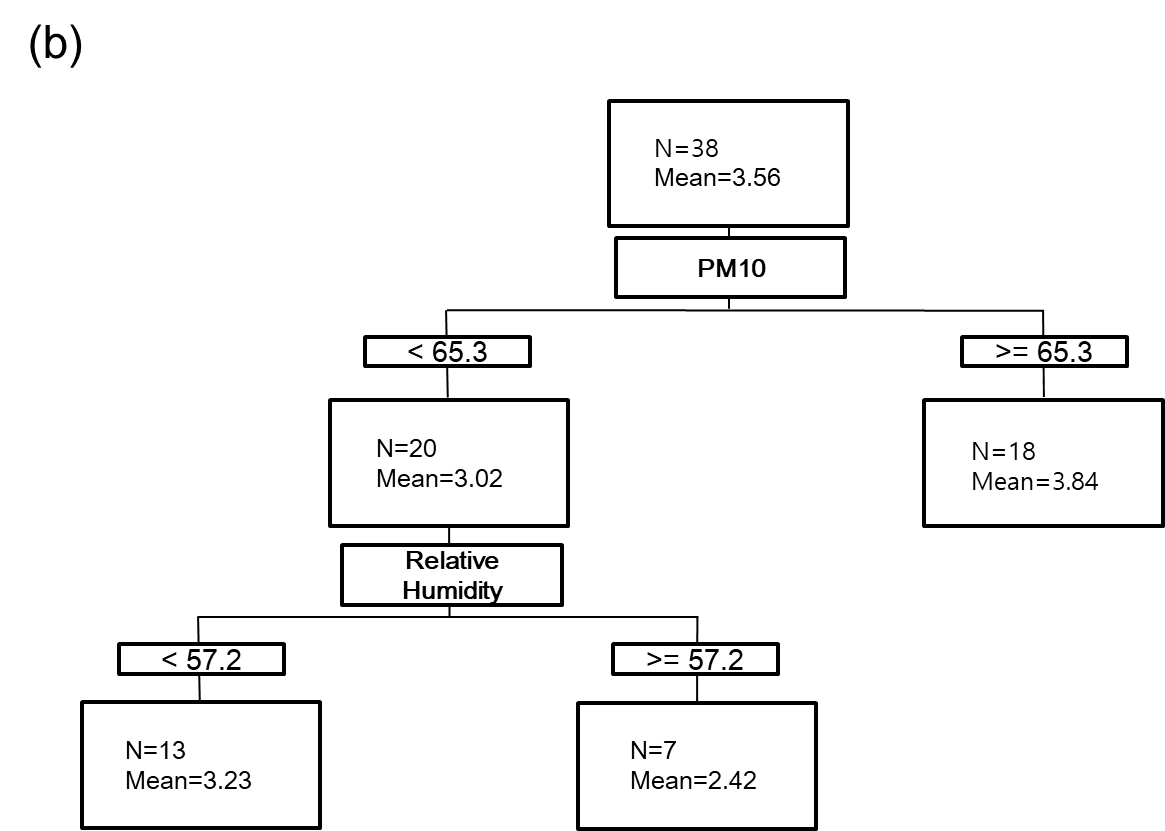


**Figure S3.** Determination of the relative importance of predictor variables in the CART model for the prediction of airborne bacterial abundance (A) and diversity (B) by binary regression tree analysis
